# Hidden Markov Model-Based Prokaryotic Genome Space Mining Reveals the Widespread Pervasiveness of Complex I and Its Potential Evolutionary Scheme

**DOI:** 10.1093/gbe/evaf154

**Published:** 2025-08-06

**Authors:** Akshay Shirsath, Snehal V Khairnar, Abhirath Anand, Divya M Prabhakaran, Amitesh Anand

**Affiliations:** Department of Biological Sciences, Tata Institute of Fundamental Research, Mumbai, Maharashtra 400005, India; Department of Biological Sciences, Tata Institute of Fundamental Research, Mumbai, Maharashtra 400005, India; Department of Biological Sciences, Tata Institute of Fundamental Research, Mumbai, Maharashtra 400005, India; Department of Biological Sciences, Tata Institute of Fundamental Research, Mumbai, Maharashtra 400005, India; Department of Biological Sciences, Tata Institute of Fundamental Research, Mumbai, Maharashtra 400005, India

**Keywords:** evolution, genomics, bioenergetics, hidden Markov model, prokaryote, Complex I

## Abstract

Most cellular reactions are interdependent; however, a subset of reactions often associate more closely to form a defined reaction pathway. An extreme arrangement of interdependent reactions occurs when the cognate proteins physically associate to constitute a complex. Respiratory Complex I (C-I) is one of the largest membrane-resident protein assemblies. Besides being a hallmark of bioenergetics, this enzyme complex is critical for maintaining redox homeostasis and facilitating transport. However, its evolutionary origins are unclear due to challenges in identifying close homologs and subunit ancestry. Using custom hidden Markov model (HMM) profiles, we analyzed the prokaryotic genome space to trace the distribution of 14 core C-I or NADH-quinone oxidoreductase (Nuo) subunits. Our findings include (i) a sensitive HMMER-based workflow for comprehensively annotating and analyzing the Nuo subunits, adaptable for similar analyses; (ii) the first species-level distribution of Nuo subunits; (iii) multiple C-I variants across ∼11,000 species, with 51.2% having a complete complex; (iv) C-I variants on plasmids, aiding evolutionary spread; and (v) extending our workflow to study mitochondrial C-I accessory subunits in prokaryotes, revealing their evolutionary roots. We also developed a web application to share our resources. Together, we comprehensively account for the distribution and probable evolutionary scheme of C-I subunits among prokaryotes.

Significance The evolution of metabolic complexity has enhanced physiological robustness and adaptive traits. The assembly of multiple functionally distinct protein subunits constituting respiratory Complex I (C-I) is an evolutionary marvel. Bacterial C-I consists of 14 subunits and is a hallmark of the electron transport system. C-I is critical for energy generation, redox homeostasis, metabolite transport, and motility. We developed an advanced statistical model and clustering-based approach to explore how the core subunits of C-I are distributed across prokaryotes. Such detailed and precise knowledge of the pathogen's metabolic capacity is critical for devising therapeutics. Beyond generating fundamental insights, we provide an adaptable workflow that enables delineation of close homologs and associated ancestry challenges.

## Introduction

NADH-quinone oxidoreductase (Nuo), commonly known as Complex I (C-I), is one of the most sophisticated assemblies of proteins wherein various subunits come together to form a relay system to transfer electrons from NADH to respiratory quinones. This multistep quantum mechanical electron tunneling is achieved by a noncovalently bound flavin mononucleotide and multiple iron–sulfur clusters spread across various subunits of the N- (NADH dehydrogenase module) and Q- (quinone hydrogenase module) modules of C-I (Hinchliffe and Sazanov 2005; Gray and Winkler 2009). The placement of multiple redox centers across a distance of approximately 95 Å allows the hopping of electrons between NADH and quinones at biologically appropriate electron transfer rates. The reduction of respiratory quinones occurs at the Q-module. The peripheral N- and Q-modules interact with the membrane-embedded P-module (proton translocation module). The P-module comprises four proton channels that pump protons to the periplasmic space by coupling the reduction potential favored electron flow in the N- and Q-modules.

C-I is a key component of the electron transport system (ETS), playing a critical role in cellular respiration. Interestingly, this complex is thought to have evolved primarily to mitigate the pH stress of ancient fermentative metabolism (Alberts et al. 2002). Mutations in this complex have recently been shown to alter intracellular acidity, leading to a state of antibiotic persistence (Van den Bergh et al. 2022). Such physiological implications suggest an ancient metabolic context to C-I evolution. C-I is a highly conserved enzyme complex (Kampjut and Sazanov 2022). While mitochondrial C-I has acquired many accessory subunits, there are 14 core Nuo subunits that are well-conserved from prokaryotes to eukaryotes (Vinothkumar et al. 2014).

**Fig. 1.**
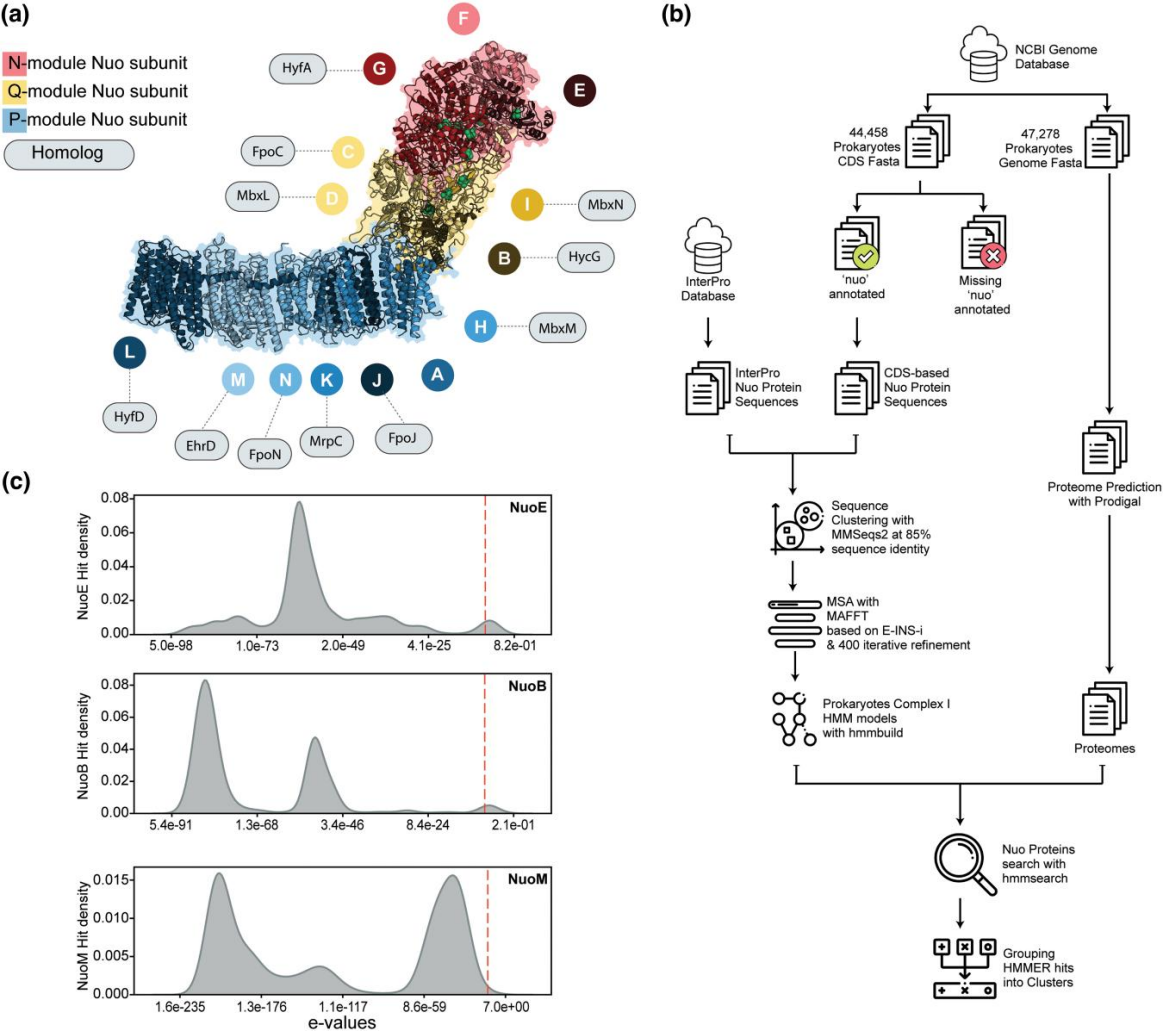
Search for the Nuo subunits in prokaryotic genomes. a) Ribbon diagram of the C-I structure from *E. coli* (PDB: 7P61) (Kravchuk et al. 2022), with functional modules: N-module for NADH oxidation, Q-module for quinone binding and electron transfer, and P-module for proton translocation. Labeled circles represent specific Nuo subunits, with a few representative homologous proteins from other complexes linked via dotted lines to indicate shared evolutionary origins or functional analogy (Table S1) (Marreiros et al. 2013). FeS clusters are shown as green spheres. b) Workflow for identifying Nuo subunits in prokaryotic genomes. Sequences from the InterPro database and annotated genomes were clustered using MMseqs2, aligned with MAFFT, and used to build HMM profiles. NuoHMMER searches identified Nuo subunit hits, which were grouped into hit clusters (Katoh et al. 2002; Eddy 2011; Steinegger and Söding 2017) c) KDE-based distributions of NuoHMMER hits. The dashed line indicates one of the generic default thresholds of 1e-7 used for selecting hits in homology-search studies (Gabaldón et al. 2005).

The modules' specific evolutionary history and their potential integration as a respiratory complex have led to the hypothesis that C-I developed in a stepwise manner from an extinct system (Schut et al. 2016; Yu et al. 2021, 2018). C-I shares an evolutionary background with several protein complexes, most importantly multiple resistance and pH adaptation (Mrp) cation/proton antiporters and membrane-bound NiFe hydrogenases (MBHs) (Fig. 1a; Table S1) (Friedrich and Scheide 2000; Marreiros et al. 2013; Ito et al. 2017). The subunits of the membrane-integrated P-module of C-I are homologous to Mrp antiporter subunits, which are both responsible for ion transport. NuoL, NuoM, and NuoN are believed to be products of gene duplication and share homology with MrpA and MrpD (Marreiros et al. 2013; Schut et al. 2016; Ito et al. 2017). The peripheral N- and Q-modules have high structural and sequence similarity with MBHs, including subunits from complexes such as Ech (energy-converting hydrogenases) and Hyc (formate hydrogenlyase). Notably, MBHs use a redox-active (NiFe) center and catalyze ferredoxin-dependent reduction of protons to molecular hydrogen. MBHs lack the coordinated long-range coupling of electron transfer and proton pumping mechanism central to C-I's function in respiratory energy transduction. Another redox-driven proton pump closely related to C-I is F_420_H_2_ dehydrogenase (Fpo complex), commonly found in methanogenic archaea. This complex couples electron transfer from the F_420_H_2_ cofactor to phenazines with proton translocation (Welte and Deppenmeier 2014). Although archaea are reported to have type II NADH:quinone oxidoreductase, the presence of NADH oxidation to proton transport coupling C-I is a subject of active research (Schäfer 2004; Jay et al. 2018).

In the present study, we focus our analysis on the canonical prokaryotic C-I (Nuo), characterized by its 14 core subunits (NuoA–NuoN), capable of catalyzing NADH oxidation coupled to quinone reduction and proton translocation. The resolution and scope of earlier research on the distribution of Nuo subunits were limited by the methodological sensitivity and limited availability of genome sequences (Gabaldón et al. 2005; Kuzniar et al. 2008; Moparthi and Hägerhäll 2011; Spero et al. 2015). The presence of homologs with high sequence and structural similarity confounds the search for valid Nuo subunits. The structural and functional diversity of individual subunits further makes generic search approaches inappropriate. We developed subunit-specific hidden Markov model (HMM) profiles for a precise search for Nuo subunits across the entire prokaryotic genome space. We clustered the highly similar sequences to avoid biasing our probabilistic model to more widely studied, and consequently genome-sequenced, prokaryotic groups. Furthermore, we used the concept that cotranscribed and functionally related genes tend to group together on chromosomes to set a subunit-specific filter cutoff. We are leveraging the genome sequence dataset on the NCBI genome database to perform a large-scale C-I subunit distribution analysis.

## Results and Discussion

### Prokaryotic Genome Dataset Curation

The advent of high-throughput sequencing and associated technological advancements has created a wealth of high-value genomics data. There were 729,850 prokaryotic genome records available on the NCBI Genome database as of September 2024 (Sayers et al. 2024). We retrieved these genomes and downloaded 47,278 genome sequence fasta files along with their associated 44,458 nucleotide coding sequences (CDSs) by applying the filtering criteria of assembly level “complete’ or “chromosome’ (Fig. 1b; Sheet S1). These genomes have satisfactory N50 values, giving high confidence in the genome assembly (Sheet S1) (Magoc et al. 2013). In our study, we sought a clear resolution of information at various taxonomic levels, such as strain and species. To obtain the corresponding taxonomy rankings, we used the NCBI taxonomy database to retrieve information for 47,278 genome records through Biopython (Cock et al. 2009; Schoch et al. 2020). Subsequently, we employed TaxonKit v0.16.0 to acquire comprehensive, hierarchical taxonomic information for each taxid included in the genome metadata (Shen and Ren 2021). After this initial retrieval and processing, our dataset comprised 10,608 unique bacterial species, represented by their 46,702 strain genomes, and 431 archaeal species, represented by 576 strain genomes.

The selected genomes were further refined based on specific taxonomic criteria to ensure consistency and reliability in downstream analyses. Genomes without designated phylum and genus classifications were excluded from the dataset, as were those marked with “Candidatus” status. This filtering step helped to ensure that the genomes included in our study have a consistent taxonomic nomenclature and a uniform structure for detailed analyses.

### Search for Potential Prokaryotic Nuo Subunits

There is a heavy bias toward some bacterial species in research, with only five species accounting for more than 40% of research papers (Jensen 2025). In our genome dataset, we observed a similar skewness with 28% of genomes representing only ten bacterial species (Sheet S2). We took a sequence clustering approach to avoid any bias in our profile-building (Fig. 1b). We extracted sequences of all 14 Nuo subunits from the CDS fasta files with existing annotations for *nuo* genes and translated them into corresponding protein sequences. These sequences were supplemented with reviewed Nuo subunit protein sequences retrieved from the InterPro database (Blum et al. 2025). By performing sequence clustering on the combined CDS and InterPro sequence pool at an 85% identity cutoff, we eliminated taxonomic overrepresentation while maintaining a maximally diverse subset for downstream HMM training.

We used the sequences obtained for each subunit to create a position-specific probabilistic model using HMMER (Potter et al. 2018). We, thereby, obtained prokaryotic subunit-specific representative HMM profiles for searching Nuo subunits. To eliminate inconsistencies in gene-calling that may arise from heterogeneous annotation pipelines, we predicted proteomes of all 47,278 prokaryotic genomes using Prodigal (Hyatt et al. 2010; Dimonaco et al. 2022). We then performed a search for Nuo subunits by applying our custom HMM profiles to these proteomes. We obtained 1,078,643 cumulative hits for Nuo subunits, of which 6,649 hits came from plasmids.

We examined the distribution of HMMER-derived e-values for each Nuo subunit hit, employing kernel density estimates (KDEs) to visualize how confidently each hit aligns with the search profiles (Scott 2018). We observed a multimodal distribution of hits for each Nuo subunit (Fig. 1c; Fig. S1). Modes with lower e-values can be assumed to consist of high-confidence hits; however, we need further support to set the e-value cutoff, as the default threshold could have included several other modes as well (Gabaldón et al. 2005).

### Identification of High-Confidence Nuo Subunit Hits

The extensive sharing of ancestry among functionally similar proteins results in sequence and structural overlap, which can pose a risk of false positives when using homology-based search tools, particularly those that apply permissive cutoffs (Rembeza and Engqvist 2021; Cremers et al. 2022). Due to homology with other aforementioned proteins, Nuo subunit hits are highly susceptible to such anomalies (Ito et al. 2017; Greening et al. 2024).

**Fig. 2.**
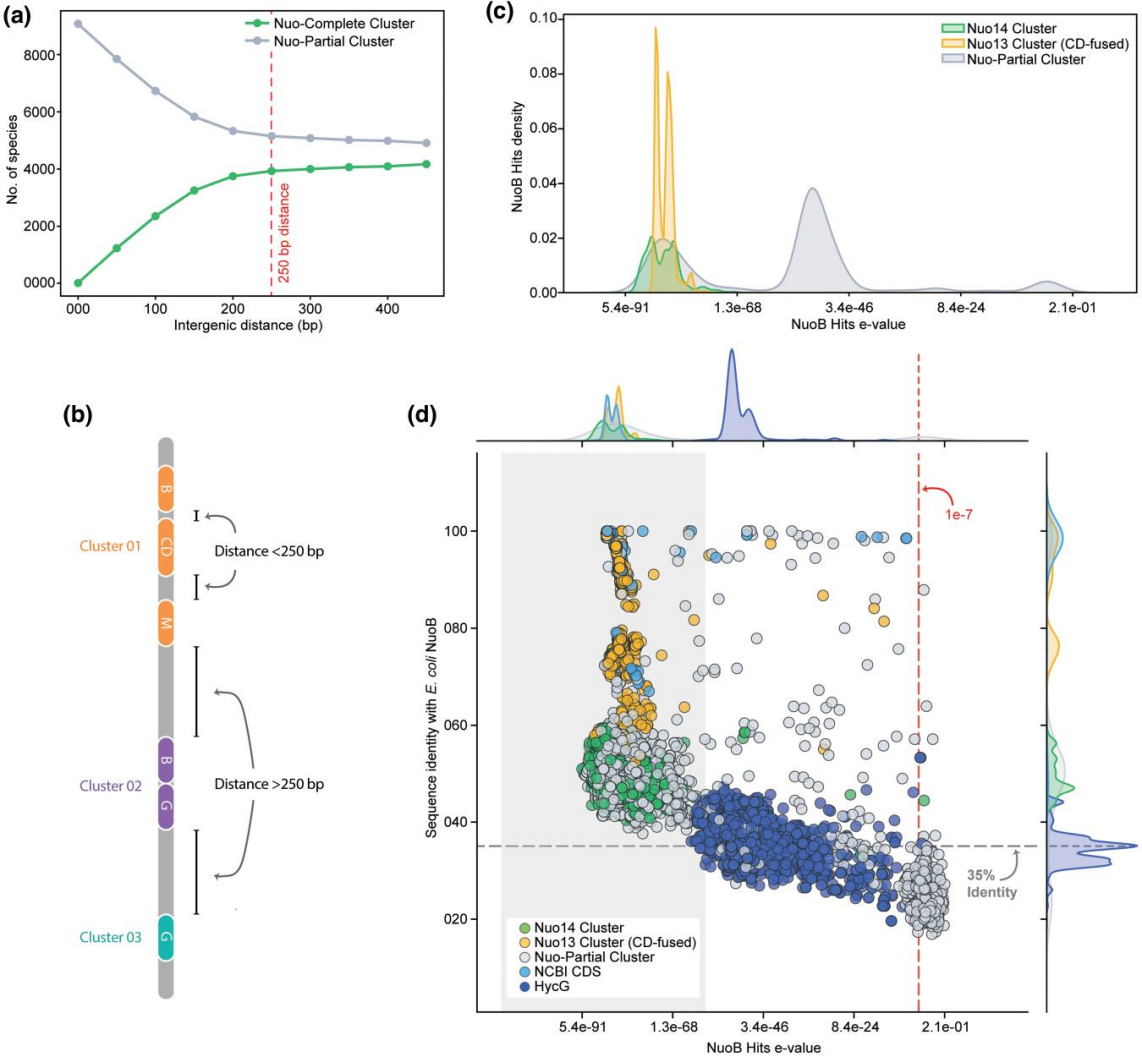
Identification of high-confidence Nuo subunit hits and characterization of NuoB subunit hits. a) Influence of maximum allowed intergenic distance for hit clustering on the number of species assigned to the Nuo-Complete cluster and Nuo-Partial cluster. The 250 bp threshold was selected to optimize gene grouping into meaningful clusters. b) Schematic representation of hits clustering based on intergenic distance. Protein subunit hits (their respective genes) within 250 bp are assigned to the same cluster (Cluster 01), while those separated by more than 250 bp are assigned to distinct clusters (e.g. Cluster 02 and Cluster 03). c) KDE-based distributions of NuoB subunit search hits' e-values. Hits belonging to the Nuo14 cluster and Nuo13 cluster (with fused CD) are highlighted. d) Scatter plot of NuoB subunit hits showing sequence identity with *E. coli* NuoB sequence (*UniProt*: P0AFC7) against e-values. NuoB subunit hits are color-coded as explained in the inset. The shaded region represents hits filtered based on the e-value threshold obtained from Nuo hits clusters analysis. Marginal density plots illustrate the distribution of e-values (top) and sequence identity (right).

The genes belonging to the same physiological functions tend to cluster together in prokaryotic genomes (Salgado et al. 2000; Osbourn and Field 2009). We chose to rely on the convergence in physiological function and the consequent grouping of the Nuo subunits to obtain high-confidence hits (Oppermann et al. 2022). We developed hit clustering criteria for grouping the Nuo subunit hits based on their respective intergenic distances across all species. Furthermore, for the hits to be grouped together, they were required to be present on the same strand. We obtained two types of hit clusters: (i) Nuo-Complete cluster containing all 14 subunit hits, i.e. NuoA-N, and (ii) Nuo-Partial cluster lacking one or more subunit(s). Nuo-Complete cluster also included clusters with fused subunits as long as all 14 subunits were obtained. As expected, we observed an initial linear increase in the number of species with the Nuo-Complete cluster as we relaxed the intergenic distance criteria for Nuo subunit hits grouping, whereas a more stringent cutoff pushed many of them into the Nuo-Partial category (Fig. 2a). However, the increase in the number of species began to level off beyond 100 base pairs and almost plateaued at an intergenic distance of 250 base pairs. Thus, we set a limit of a maximum of 250 base pairs separation between hits to be grouped in a cluster (Fig. 2b). It is noteworthy that our hit-clustering parameter may or may not follow the classical operon definition due to a lack of empirical data.

We categorized the hits on the e-value KDE plots according to their clustering pattern. We further segregated the Nuo-Complete cluster into (i) the Nuo14 cluster and (ii) the Nuo13 cluster with fused C and D subunits (Fig. 2c; Fig. S2). We observed significantly lower e-values for Nuo hits found in these hit clusters, and these hits aligned with the left-most mode on the KDE plot. This distinction in segregation allowed us to define e-value cutoffs specific to each Nuo subunit. The presence of fused variants (e.g. NuoCD) underscores the pipeline's ability to handle nontraditional gene structures. Even in cases where canonical gene boundaries are altered, the established e-value thresholds remain effective, indicating that HMM profiles are robust tools for recognizing modified arrangements. Overall, the lower e-values and an empirical setting of the cutoff allowed us to remove low-confidence hits (Table S2).

We further analyzed the hits by plotting their protein length against the e-value. It was gratifying to note that the Nuo hits arranged in clusters showed a length distribution within the range coinciding with UniProt for the corresponding subunits (Fig. S3) (UniProt Consortium 2025). This observation allowed us to eliminate outliers based on their unusual protein length. From a broader perspective, the e-value-based filtering strategy complements the earlier genomic context and clustering analyses. While the operon structure, defined by parameters such as intergenic distance and strand orientation, provides a genomic framework, the KDE distributions offer a sequence-level measure of confidence.

We also compared the protein sequence identity of the hits with the corresponding subunit from *Escherichia coli*. Although we did not use sequence identity as a filtering criterion, comparing e-values with sequence identity relative to *E. coli* reinforces the robustness of our approach (Fig. 2d). Nuo-Complete clusters, which yield low e-values under our KDE-based thresholds (Fig. 2d, gray region), also tend to exhibit higher sequence identity. Notably, COG classified the majority of the NuoB HMMER hits with poor e-value as the HycG subunit of the formate hydrogenlyase complex (Galperin et al. 2019). HycG shares an evolutionary link with NuoB (McDowall et al. 2014). The segregation of distant homologs based on our custom e-value thresholding substantiates the sensitivity of our HMMER-driven approach to searching high-confidence Nuo subunits.

### Distribution and Coverage of Nuo Subunits

**Fig. 3.**
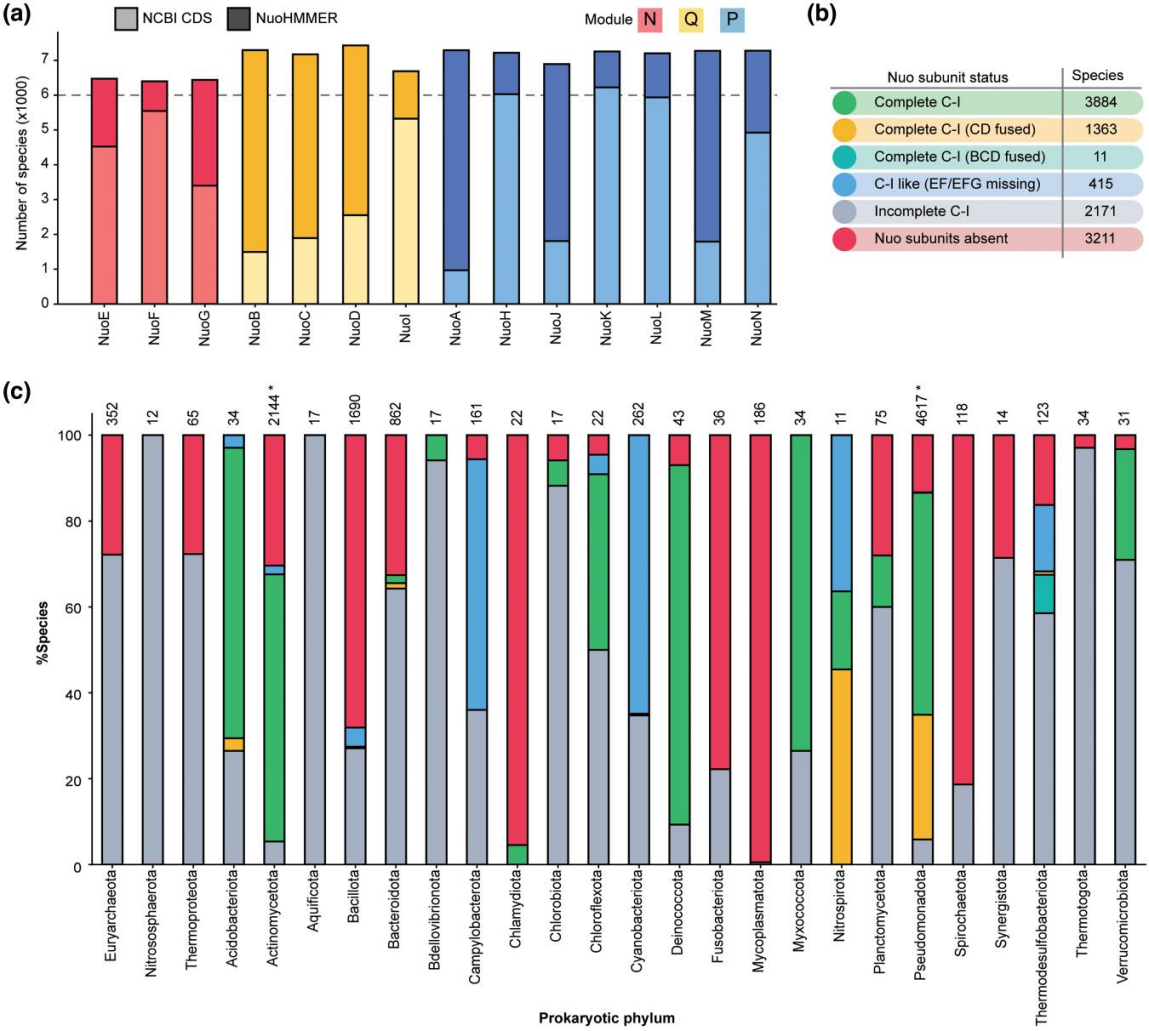
Distribution of Nuo subunits across prokaryotic species. a) Stacked bar chart showing the number of species with Nuo subunits identified by NuoHMMER and existing NCBI CDS annotations. Bars are color-coded by functional modules. b) Species abundance for C-I variants based on Nuo subunit status. c) Percentage distribution of species with different Nuo subunit variations across various phyla. The first three phyla belong to archaea (from left). Bar segments are color-coded to indicate specific subunit variations (refer to b)). The asterisk represents phyla where a species shows two different C-I variants. Phyla with fewer than ten species have been excluded from the plot to avoid any representative bias.

Despite the widespread conservation of C-I, our understanding is restricted to a few commonly studied model species (Baradaran et al. 2013; Kolata and Efremov 2021; Liang et al. 2023; Ivanov et al. 2024). An earlier phylogenomics-based study of 970 bacterial genomes reported the presence of complete C-I in 509 genomes (Spero et al. 2015). Our species-level taxonomy ranking obtained for the 46,702 strain genomes allowed us to examine the distribution of Nuo subunits among 10,608 unique bacterial species. The knowledge gap filled by us brought uniformity in the distribution of various Nuo subunits (Fig. 3a; Sheet S3). The existing NCBI genome annotation for the Nuo subunits shows that complete C-I is present in 418 species. Remarkably, our search expanded the coverage of complete C-I by almost 10-fold (Fig. 3b). In 1,374 of the species with complete C-I, subunit D is fused either with subunit(s) C or with both B and C. We found two complete sets of Nuo subunits in 45 species. *Rhodopseudomonas palustris* is reported to harbor two of these complexes to diversify its metabolism (Pechter et al. 2016). Another species where we found two complete C-I was *Acidiphilium cryptum*. *Acidiphilium* spp. survive extreme conditions, and an abundance of laterally acquired genes are believed to be responsible for their adaptive metabolic expansion (Li et al. 2020). The presence of two NADH-driven proton efflux systems could be responsible for their acidophilic nature.

A notable group includes 415 species in which this enzyme is classified as “C-I-like,” because of missing EF or EFG subunits. These subunits constitute the N-module that harbors the FMN cofactor and is responsible for the oxidation of NADH. Such configurations suggest adaptations to specific ecological niches, where the absence of this module may reflect a variation of C-I to support alternative respiratory strategies. The incomplete C-I category, comprising 2,171 species, represents species where only subsets of Nuo subunits were detected. Some incomplete configurations might have resulted from genomic fragmentation or assembly artifacts. Lastly, 3,211 species lacked Nuo subunits entirely, suggesting a reliance on alternative respiratory pathways or metabolic strategies.

Our dataset consisted of genomes belonging to multiple strains of a species. We, therefore, examined the consistency of C-I variants within a species. We observed consistent C-I variation in all but one species (Sheet S4). *Stenotrophomonas rhizophila* has complete C-I with 14 subunits, whereas one strain has 13 subunits with fused CD. This intraspecies variant conservation encouraged us to examine the C-I variant distribution at a higher taxonomic rank. We examined the distribution of C-I variants within every phylum.

We observed the presence of Nuo subunits in three archaeal phyla encompassing seven classes (Fig. 3c; Table S3). Halobacteria and Methanomicrobia showed a C-I-like variant that lacks the N-module. However, our stringent filtering parameter eliminated the subunits I and J from these two classes. Methanomicrobial species *Methanosarcina mazei* complements the N-module deficiency by alternative electron input from the FpoF-mediated oxidation of a lower potential electron carrier F_420_H_2_ (Baumer et al. 2000; Friedrich and Scheide 2000). While some of the Nuo hits showed a significant domain similarity with F_420_H_2_ dehydrogenase subunits, the hits obtained for P-module subunits NuoA, NuoM, and NuoN retained their canonical Nuo subunit identity. This subunit arrangement was observed in 13 species of Methanomicrobia, suggesting a widespread use of cofactor F_420_H_2_ as the electron donor in this class. These observations strengthen the proposed course of evolution of C-I, where P–Q-modules are initially believed to have assembled, and later, bacteria acquire N-modules to exploit higher potential electron carriers (Schut et al. 2016; Yu et al. 2021). Notably, the coupling efficiencies achieved using NADH as a cofactor are significantly higher compared to F420 (Baumer et al. 2000).

Bacterial phyla showed a varied distribution pattern for the C-I variant (Fig. 3c). Actinomycetota and Pseudomonadota, making up around 64% of the total bacterial species in our dataset, showed several variants of C-I with a higher prevalence of the complete C-I. In Acidobacteriota, most species showed complete C-I, which may be related to their acid tolerance, given the hypothesis that pH homeostasis was the selection pressure for the evolution of ATP-independent proton transporters (Alberts et al. 2002; Kalam et al. 2020). We found several bacterial phyla where Nuo subunits were mostly lacking. These phyla include Bacillota (with 16% of the total bacterial species in our search), Mycoplasmatota, Spirochaetota, Chlamydiota, Fusobacteriota, etc. Mycoplasmatota and Spirochaetota consist of bacterial species that are obligately dependent on hosts (Stülke et al. 2009). *Mycoplasma pneumoniae* is known to lack a functional respiratory chain and rely on organic acid fermentation for ATP supply (Wodke et al. 2013). Similarly, species from Chlamydiota are also reported to depend on the host for their metabolic energy requirements (Peeling and Brunham 1996; Liang et al. 2018).

We have an interesting observation from Thermodesulfobacteriota, which are believed to be anaerobic (Mori 2014). This phylum has ten distinct classes, out of which four classes (Desulfuromonadia, Desulfobacteria, Desulfovibrionia, and Desulfarculia) showed a distinct complete C-I variant consisting of fused BCD subunits (Table S4). Desulfobacterial species inhabit cyanobacterial microbial mats, resulting in toxic exposure to oxygen, and their oxidative stress defense strategies involve reduction of oxygen using cytochrome *bd* oxidases (Schnaars et al. 2021). While this oxidase is suggested to mitigate the load of reactive oxygen species, cytochrome *bd* oxidases are often part of aerobic ETS (Patil et al. 2024). Notably, Thermosulfobacteria can survive suboxic conditions, and our observation of complete C-I in this class raises the possibility of adaptive oxic energy metabolism (Inskeep et al. 2025).

### Association of C-I Variants With Taxa and Metabolic Lifestyles

**Fig. 4.**
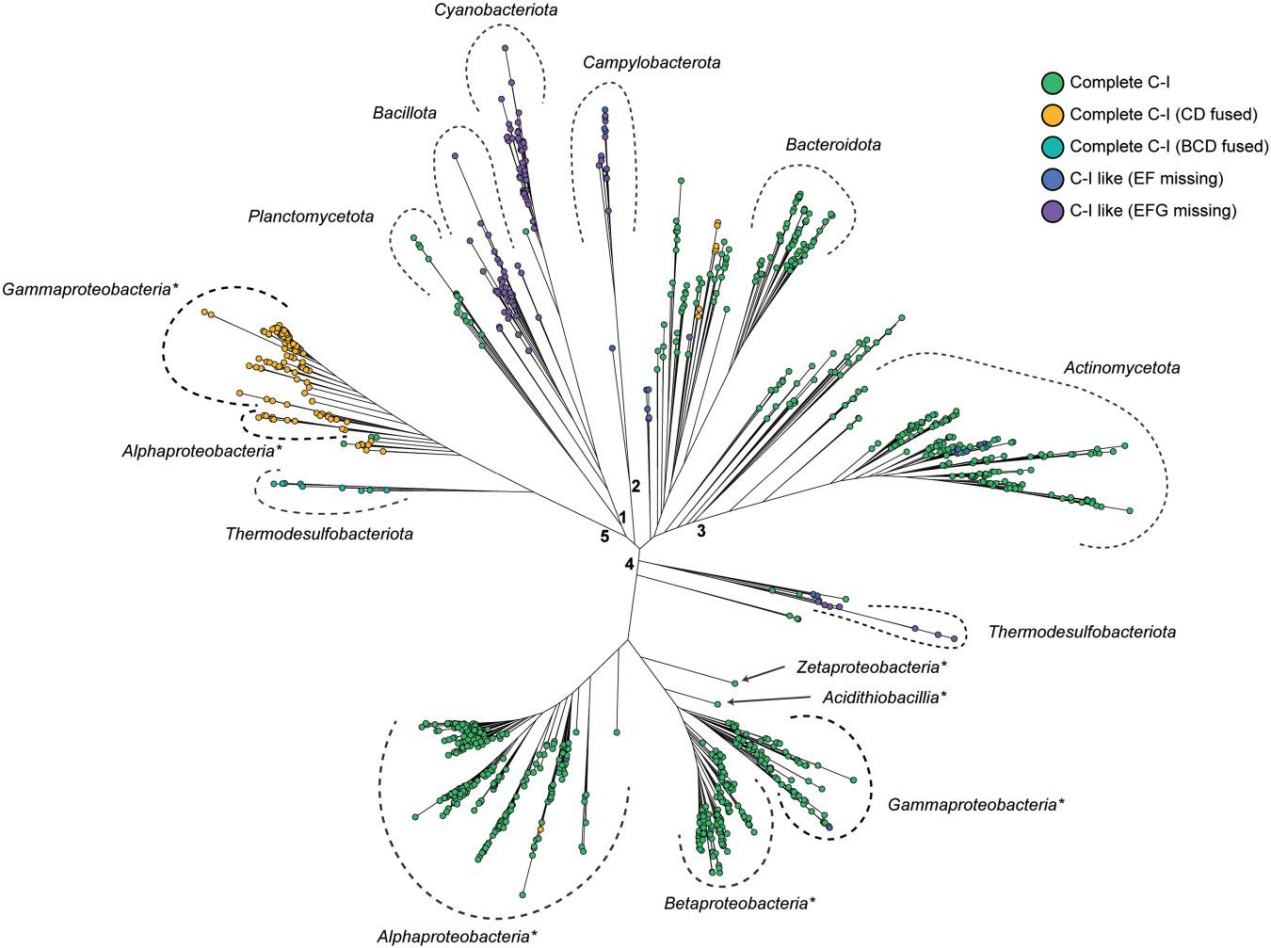
Association of C-I variants with taxa. The ML-based phylogenetic tree is based on concatenated sequences of Nuo subunits from one species per genus. The C-I variations have been shown by color-filled circles. Bacterial taxonomic phyla and classes (marked with an asterisk) have been highlighted using curved dashed lines.

We further focused on the association of complete C-I variants with various prokaryotic taxa. A total of 5,258 bacterial species, encompassing 1,196 genera, have all 14 Nuo subunits. We used concatenated sequences of all Nuo subunits to construct a phylogenetic tree based on the maximum likelihood (ML) principle (Fig. 4). We observed five major phyletic clades. In clade 2, we observed a C-I-like variant to form a monophyletic distribution of Campylobacterota, suggesting an overall sequence diversity compared to other variants. We observed two distinct subclades in clade 1. One of these two subclades consists of C-I-like variants coming from ancient bacterial phyla, Bacillota and Cyanobacteriota. Cyanobacteriota is believed to have functional C-I, but the identity of the N-module supplement is under investigation (Friedrich and Scheide 2000). The majority of the clades have a mix of split (Nuo subunits not clustered together) and single cluster C-I; however, the subclades occupied by Actinomycetota in clade 3 and Gammaproteobacteria and Betaproteobacteria in clade 4 are dominated by single cluster C-I (Fig. S4). The clades 4 and 5 showed polyphyletic distribution with species from Gammaproteobacteria and Alphaproteobacteria split among them. Both these clades consist of complete C-I variants, with clade 5 populated by variants with fused C and D subunits.

**Fig. 5.**
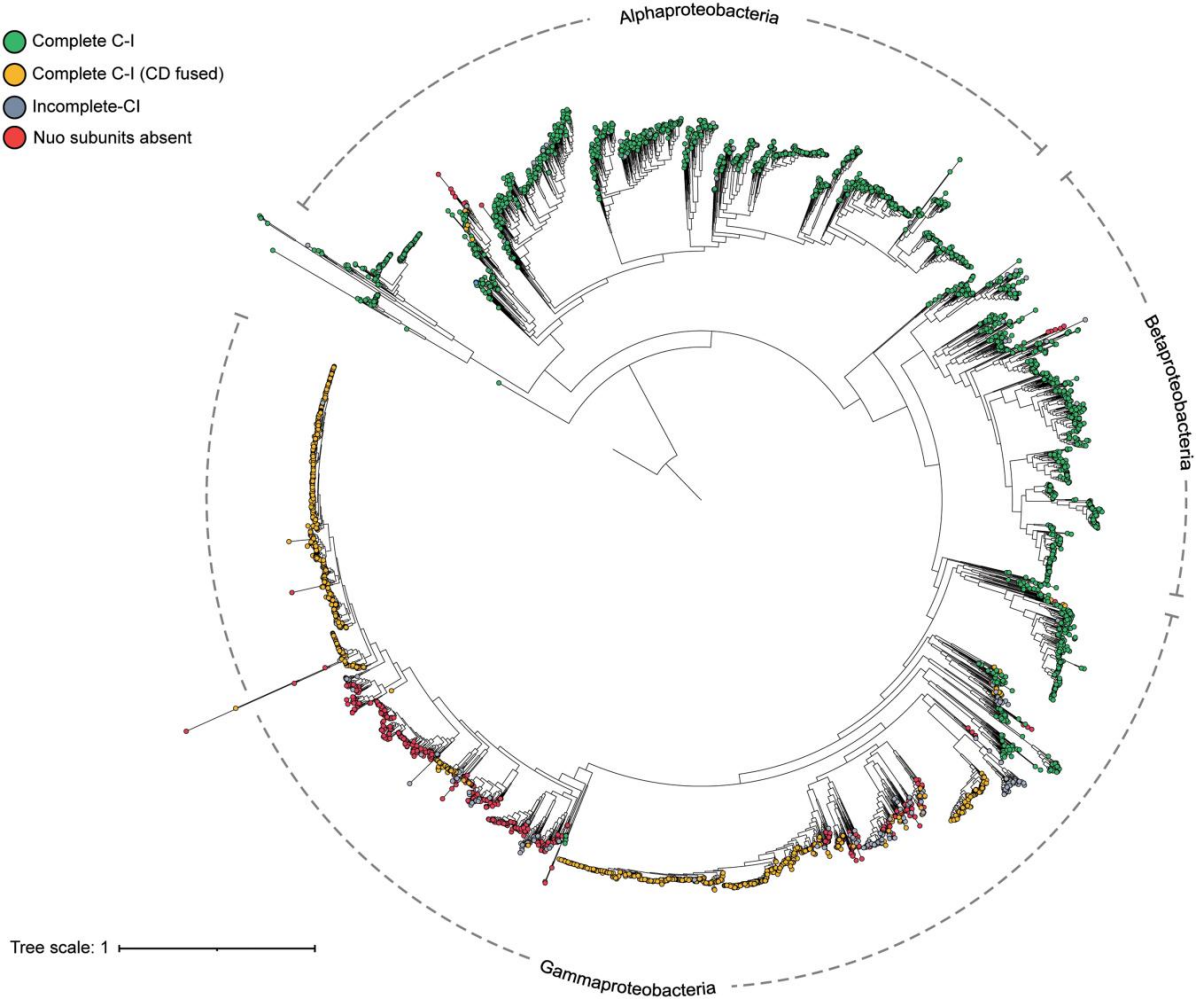
Phylogenetic tree of Pseudomonadota showing C-I variants. GToTree-based circular phylogenetic tree of Pseudomonadota rooted with *A. capsulatum*. Branch lengths are proportional to evolutionary distance; the scale bar measures divergence. Dashed arcs divide the three major taxonomic classes—Alphaproteobacteria, Betaproteobacteria, and Gammaproteobacteria—and their tips have colored symbols indicating the respiratory C-I variants.

The splitting of gammaproteobacterial species into two separate clades motivated us to look at the evolutionary history of the relevant C-I variants. To achieve this, we overlaid C-I variant data onto a rooted phylogenetic tree of Pseudomonadota species (Fig. 5). The phylogenomic timeline of proteobacterial divergence is reflected in the tree structure. Alphaproteobacteria first appeared around 1.9 billion years ago, followed by Betaproteobacteria and Gammaproteobacteria as the most derived subgroups (Degli Esposti 2014; Wang and Luo 2021).

Upon layering the C-I variant information on this species tree, we observed a distinctive pattern. The evolutionarily older species showed the presence of 14 subunits’ complete C-I, whereas modern species have 13 subunits’ complete C-I variant with C and D subunits fused. Functional synergy is often a cause of gene fusion, and modern variants with fused subunits support the evolutionary convergence of distinct proteins to achieve a collective physiological function (Bolotin et al. 2023).

C-I reduces the respiratory quinones, which can then interact with either the oxic or anoxic components of the ETS based on the metabolic demand. We were motivated to examine the oxygen-dependent metabolic lifestyle preferences of bacterial species with complete C-I. We collected lifestyle information from the BacDive and IMG databases (Sheet S5) (Chen et al. 2019; Reimer et al. 2022). We also performed an extensive literature survey to fetch information for species that were missing from the above databases.

**Fig. 6.**
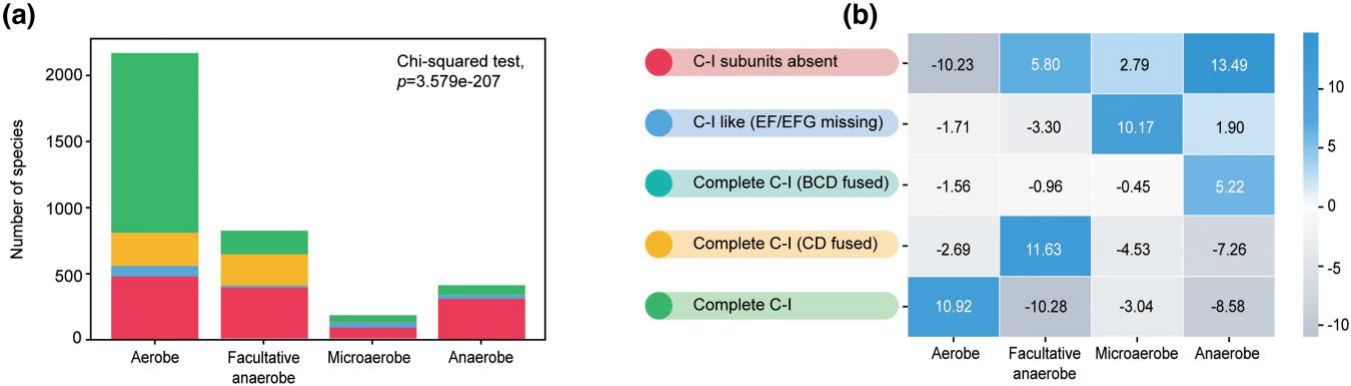
Association of C-I variants with metabolic lifestyles. a) Distribution of species with various metabolic lifestyles (aerobe, anaerobe, facultative anaerobe, and microaerobe) based on C-I variations. The statistical significance of the relationship between oxygen tolerance and C-I status was assessed using a chi-square test. b) Heatmap showing residuals from the chi-square test, representing deviations from expected distributions of C-I variants for a given metabolic lifestyle. Positive values indicate an association, while negative values indicate dissociation.

We observed a significant positive association between the aerobic nature of the bacteria and the presence of complete C-I (Fig. 6a, and b). Interestingly, obligate aerobes and facultative anaerobes showed distinct C-I variants. A similar association was reported earlier (Spero et al. 2015). It is gratifying that the lifestyle association derived from a smaller dataset of 750 species remains valid for a five times larger dataset. We also observed microaerophilic bacteria to associate strongly with Nuo-like C-I. This group includes *Campylobacter jejuni*, which is known to lack a functional N-module and rely on flavodoxin as the source of electrons (Weerakoon and Olson 2008). Interestingly, the complete C-I variant with fused BCD subunits showed an association with an anaerobic lifestyle. As discussed in the previous section, the Thermodesulfobacteriota species possessing this C-I variation likely have minimal oxic metabolism.

### Nuo Subunits on Plasmids

The widespread distribution of Nuo subunits and various C-I variants makes it appealing to assume that Nuo subunit genes traveled among prokaryotic species, resulting in the assembly of complete C-I (Harrison and Brockhurst 2012). Typically, such gene transfer events are mediated by plasmids, which carry genes that assist their host in acquiring adaptive traits (Salgado et al. 2000).

**Fig. 7.**
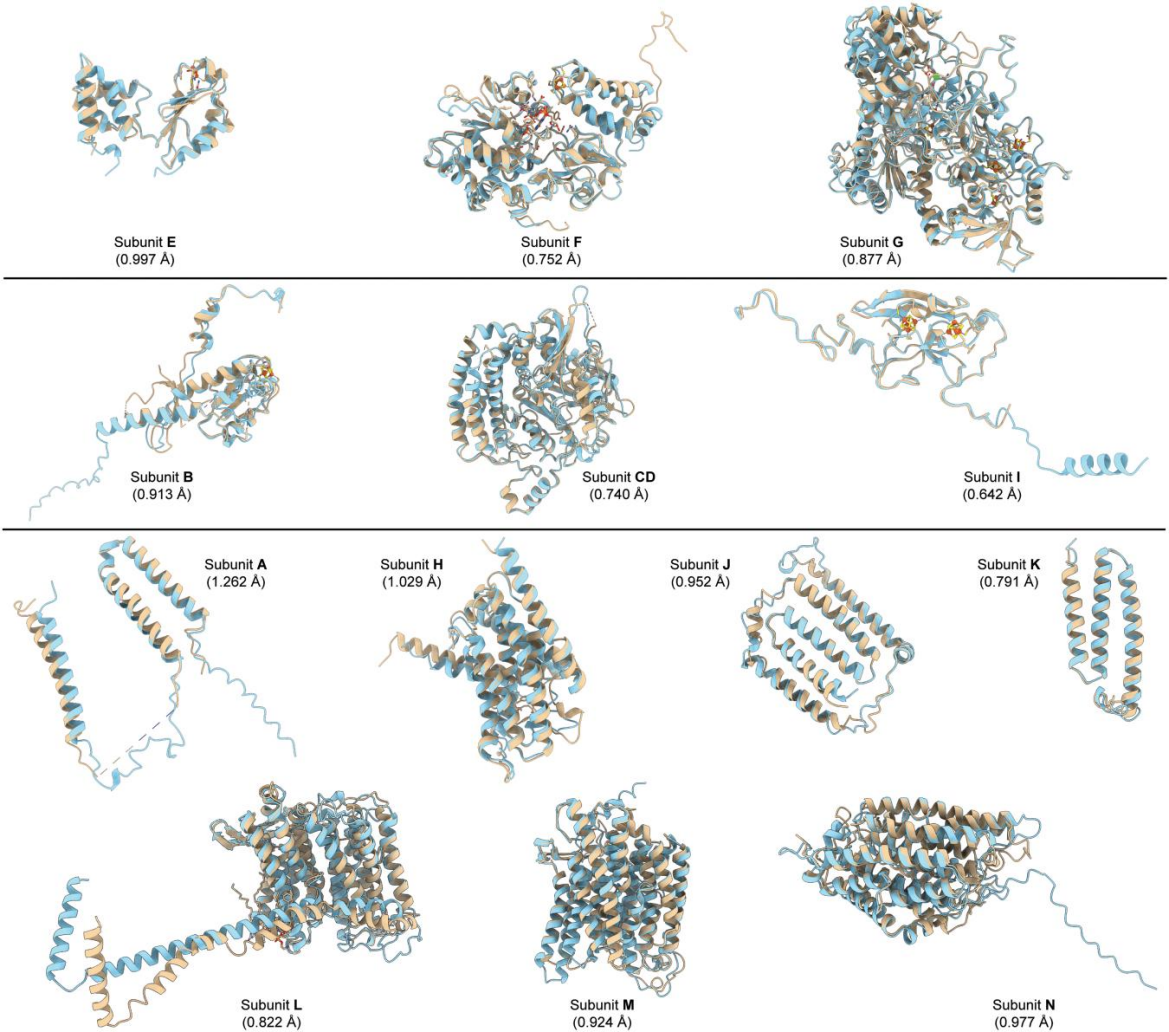
Structural comparison of Nuo subunits from the plasmid. The alignment of cryo-EM structures of *E. coli* Nuo subunits (chromosomal origin, PDB: 7P61, beige) with the corresponding AlphaFold3-predicted structure for plasmid-derived hits in *K. saccharivorans* (blue). Their respective RMSD values are shown in brackets.

Our dataset enabled us to distinguish between Nuo subunit hits originating from chromosomes and plasmids. We noted that Nuo subunits were located on plasmids in 57 species (Sheet S6). While in most cases, only a partial set of Nuo subunits was obtained, 17 species showed a complete set of Nuo subunits on plasmids (Table S5). Interestingly, Nuo subunits were exclusively located on plasmids in five bacterial species. This genomic distribution of Nuo subunits has never been reported; therefore, we performed further validation for this observation. We picked the plasmid-borne Nuo subunit sequences from *Komagataeibacter saccharivorans* and built protein structures for individual subunits using AlphaFold3 (Abramson et al. 2024). Then we examined their structural conservation with cryo-EM-derived structures of chromosomally located *E. coli* Nuo subunits (Fig. 7). We obtained very high structural conservation between corresponding subunits. The presence of Nuo subunits, and in a few instances complete C-I, on plasmids indicates a potential mechanism of intra- and interspecies transfer of corresponding genes.

### Mitochondrial C-I Accessory Subunits in Prokaryotes

The evolutionary assembly of multiple subunits to form the complete C-I has continued in eukaryotes, and mitochondrial C-I has acquired additional subunits to form a 45-subunit complex. These additional subunits are accessory to the core subunits, but their presence is critical for the assembly and function of human mitochondrial C-I (Stroud et al. 2016).

Interestingly, while bacterial C-I with 14 subunits is fully functional, recent studies have shown a few accessory subunits in some bacteria. The cryo-EM structure of *Mycobacterium smegmatis* C-I showed subunit A9 as part of the complex, whereas three accessory subunits (A12, S4, and S6) were reported from *Paracoccus denitrificans* (Liang et al. 2023; Ivanov et al. 2024).

**Fig. 8.**
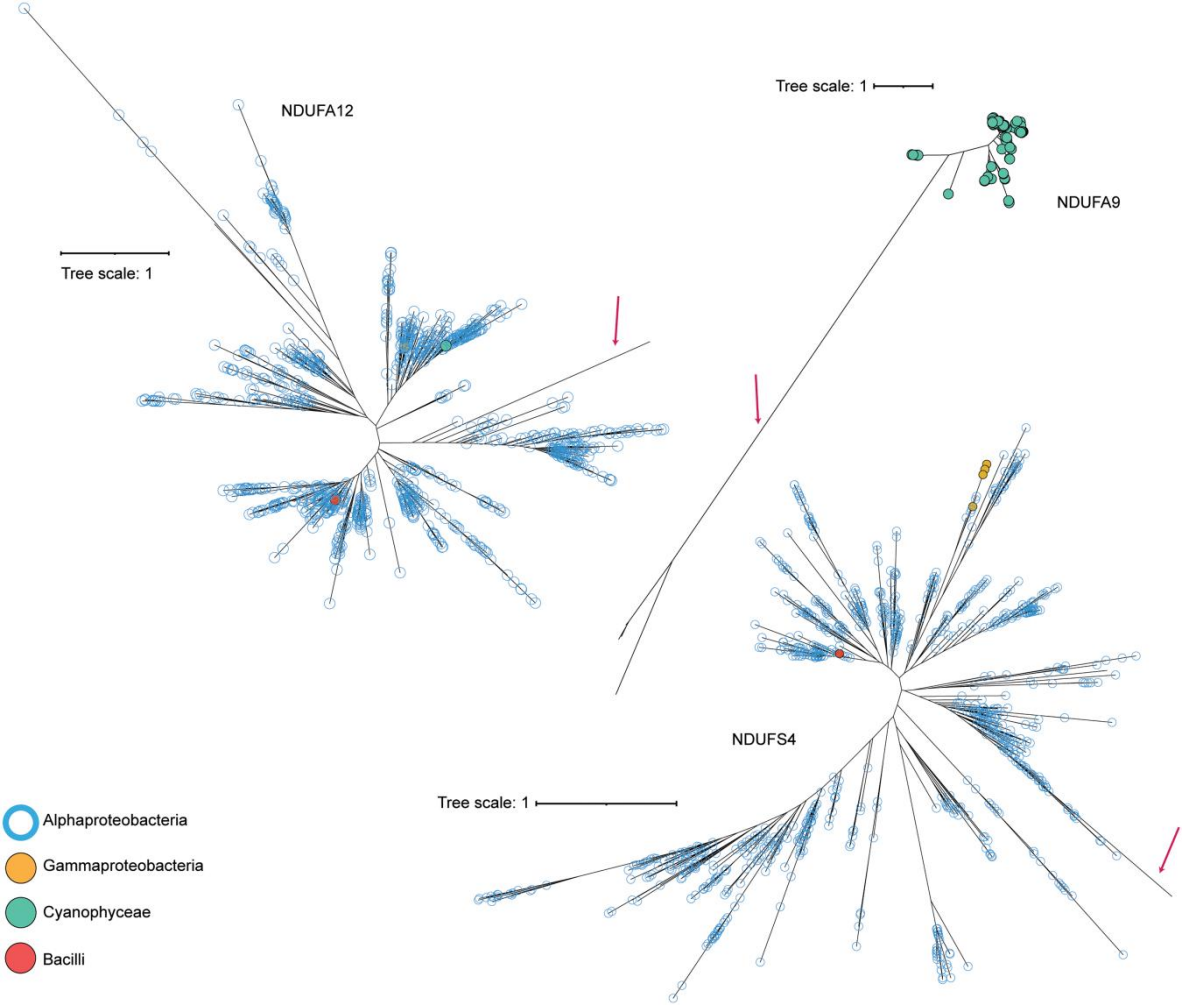
Phylogenetic tree for the C-I accessory subunits. ML-based phylogenetic trees were constructed for three accessory subunits (NDUFA12, NDUFA9, and NDUFS4) of C-I using sequences identified through the NuoHMMER workflow. The trees display bacterial species that contain matches for these subunits. Each circle represents a bacterial species, color-coded according to its respective taxonomic class. Eukaryotic sequences are marked with arrows. Branch lengths indicate evolutionary distance in terms of substitutions per site.

We, therefore, followed our workflow to probe the distribution of such accessory subunits in prokaryotes. In this case, the HMM profiles for all 31 mitochondrial accessory subunits were built using corresponding sequences from the InterPro database, which were, as expected, dominated by eukaryotic sequences. We observed hits for A9, A12, and S4 accessory subunits in 1,402 bacterial species, out of which 1,279 species belong to Alphaproteobacteria (Fig. 8; Sheet S7; Fig. S5). Notably, we have found the complete C-I variant in these alphaproteobacterial species. This close association of accessory subunits with complete C-I suggests their functional involvement. Notably, both Rhodobacterales and Rickettsiales, which are argued to be closer to the mitochondrial C-I evolution, belong to this class (Martijn et al. 2018; Jarman et al. 2021; Muñoz-Gómez et al. 2022). The subunit A9 is absent across all alphaproteobacterial species and was found localized in 115 species of Cyanophyceae. These observations suggest a more distributed evolution of C-I accessory subunits in prokaryotes, which would have converged later during the endosymbiont event.

Our study heavily relies on the quality of the genome dataset. To ensure that the genome assembly artifacts do not influence the conclusions, we reexamined the N50 values of genomes belonging to complete, incomplete, and absent C-I categories. We did not observe a significant relationship between genomes with incomplete or absent C-I and a poor N50 value (Fig. S6a and b). We also examined the percentage distribution of N50 categories within each prokaryotic phylum and observed that genome assemblies were overwhelmingly categorized as Excellent, with only rare exceptions in lower categories (Fig. S6c). Collectively, these analyses indicate that the absence or incompleteness of C-I is likely a biological feature, rather than a consequence of genome assembly quality.

We report several observations and findings that require focused experimental studies. We have developed a web application (prokcomplexone.streamlit.app) on the Streamlit platform to provide an interactive platform for researchers to explore the results of our comprehensive study on the distribution of Nuo subunits and diversity of C-I across prokaryotic genomes. This WebApp can facilitate bridging the gap between experimental and computational analyses and may potentiate translational efforts.

The assembly of various proteins with distinct biochemical functions for the evolution of C-I is a marvel of molecular innovation. Such complexation is driven by selection pressure on assembly intermediates, and evolution selects for protein complexes that assemble via ordered pathways (Marsh et al. 2013). An appealing immediate prospect of our study would be to map the Nuo subunits identified in this work onto the bacterial tree of life to trace the evolutionary course of subunits' assembly. Our observations prompt several fundamental questions that remain to be fully explored. What were the physiological roles of various C-I modules before their complete assembly? Do the C-I variants have differences in biochemical activities? Given the pleotropic significance of this enzyme complex in bacterial physiology, it would be interesting to probe how cellular energetics, redox homeostasis, and motility are supported in the species lacking C-I. Active efforts are underway to exploit bacterial energy metabolism as the target space for antibiotic development (Murima et al. 2014; Hards and Cook 2018). Given its absence from mitochondrial energetics, the nonproton–pumping alternative to C-I, type II NADH:quinone oxidoreductase is considered an effective target (Harbut et al. 2018). However, the success of such approaches critically depends on detailed and precise knowledge of the pathogen's metabolic capacity.

## Materials and Methods

### Data Acquisition

As of September 5, 2024, the NCBI Genome database contained 729,729 prokaryotic genome records. For this study, we selected 47,278 genomes classified as either “Complete Genomes” or “Chromosomes.” We classified genome assemblies based on how their N50 values compare to the total genome length and the number of contigs they contain. An assembly is deemed “Excellent’ if its N50 is at least 90% of its total length and it has no more than five contigs. It is considered “Good’ if the N50 is at least 70% and the contig count is at most ten. Assemblies with an N50 of at least 50% and up to 50 contigs are labeled “Moderate,” and any assembly that fails to meet these cutoffs is considered “Poor.” This dataset includes 46,702 bacterial genomes and 576 archaeal genomes (Sheet S1). Taxonomic rankings were retrieved using TaxonKit v0.14.0 (Shen and Ren 2021) via NCBI TaxIDs, ensuring consistent species-level resolution for downstream analyses. Our genome dataset includes 11,039 prokaryotic species.

### Nuo Subunit-Specific HMMER Profile Construction

Annotated *nuo* subunit sequences were extracted from CDS FASTA files, translated into corresponding protein sequences, and supplemented with reviewed Nuo subunit sequences from the InterPro database to ensure a comprehensive dataset (Blum et al. 2025). To reduce sequence redundancy, MMseqs2 v13.45111 was used to perform sequence clustering of Nuo proteins at 85% sequence identity (Steinegger and Söding 2017). Multiple sequence alignments (MSAs) were performed using MAFFT v7.490 (E-INS-i strategy), optimized for sequences with multiple conserved domains, and refined over 400 iterative cycles (Katoh et al. 2002). Using HMMER v3.4, we built 16 individual HMM profiles (14 for individual subunits and two for fused subunits), with hmmbuild, incorporating Laplace priors to enhance statistical robustness (Eddy 2011). For each subunit, sequence weighting was disabled (–wnone) to ensure equal representation of all sequences within that subunit's dataset, while core model definition and fragment handling were adjusted (–symfrac 0.6, –fragthresh 0.3) to balance conservation with sequence variability.

### Systematic Identification of Nuo Subunits Using HMM Profiles and Filtering

Before HMMER-based subunit detection, proteome prediction for all genome files was performed using Prodigal v2.6.3 (Hyatt et al. 2010). Subsequently, the predicted proteomes were searched against the NuoHMMER profiles through hmmsearch (HMMER v3.4). Following the HMM search, we analyzed the distribution of e-values for each Nuo subunit hit and employed KDEs to assess the distribution. To minimize false hits, we implemented a filtering strategy based on three key criteria: hits clustering, KDE-based e-value cutoffs, and protein length validation.

### Metadata Curation and Associated C-I Variant Analysis

Metadata on oxygen requirements was retrieved from the BacDive and IMG databases (Chen et al. 2019; Reimer et al. 2022). For species lacking entries in these databases, manual curation from literature sources was performed (Sheet S4). Oxygen tolerance was categorized into four groups: aerobe, anaerobe, facultative anaerobe, and microaerobe. To assess the relationship between C-I variants and oxygen tolerance, a chi-square test of independence was conducted (*P* < 0.0005). A contingency table was constructed to compare observed frequencies and statistical significance was evaluated.

### C-I Variant Phylogenetic Tree

Phylogenetic analyses were conducted using concatenated protein sequences from 1,196 bacterial species, with one species selected per genus. The selection criteria ensured that each genus included in the dataset possessed C-I and its variations. MSA was performed using MAFFT v7.490 (Katoh et al. 2002) with the L-INS-i algorithm, optimized for accuracy in large datasets. The alignment was subsequently trimmed using BMGE v1.12 (Criscuolo and Gribaldo 2010) to remove poorly aligned and compositionally biased regions. ML phylogenies were inferred using IQ-TREE v1.6.12 (Minh et al. 2020), with the best-fit substitution model determined by ModelFinder. Branch support was assessed using ultrafast bootstrap approximation (UFBoot, 1,000 replicates) and the SH-aLRT test (1,000 replicates). The final dataset consisted of 2,230 alignment positions, including 2,099 parsimony-informative sites, with automatic filtering of gapped and compositionally biased sequences. As an alternative approach, approximately-ML trees were constructed using FastTree v2.1 (Price et al. 2010) with the WAG + Gamma model, which is optimized for large datasets. The resulting phylogenies were used to investigate the evolutionary relationships of C-I variants across prokaryotic lineages.

### Pseudomonadota Phylogenetic Tree With C-I Variants

The bacterial phylogenetic tree of Pseudomonadota was produced with GToTree v1.6.3 (Lee 2019), using the prepackaged single-copy gene-set for Bacteria (74 target genes). Target genes were identified with HMMER3 v3.2.2 (Eddy 2011), individually aligned with muscle v5.1 (Edgar 2022), trimmed with trimal v1.4.rev15 (Capella-Gutiérrez et al. 2009; Edgar 2022), and concatenated prior to phylogenetic estimation with FastTree2 v2.1 (Price et al. 2010). Two hundred fifty-three genomes were omitted from the final tree because they were either duplicates or lacked a sufficient number of single-copy marker genes. The generated tree was rooted with *Acidobacterium capsulatum*.

### Plasmid-Borne Nuo Subunit Structure Prediction

The three-dimensional structures of individual subunits were predicted using AlphaFold3 (Abramson et al. 2024). The amino acid sequences of the subunits were used as input for the AlphaFold server. The highest-ranked model based on the predicted local distance difference test (pLDDT) score was selected for further analysis.

### Mitochondrial C-I Accessory Subunits HMM Profiles

To identify mitochondrial C-I accessory subunits in prokaryotic proteomes, we constructed HMM profiles following the same protocol used for Nuo subunits. Instead of extracting sequences from CDS FASTA files, we obtained protein sequences from the NCBI Protein database and InterPro database to ensure a comprehensive dataset. To reduce redundancy, sequences were clustered at 85% identity using MMseqs2. MSAs were performed with MAFFT v7.490 (E-INS-i strategy) and refined over 400 iterative cycles. HMM profiles were generated using HMMER v3.4, incorporating Laplace priors for statistical robustness. These HMM profiles were subsequently used for systematic detection of mitochondrial C-I accessory subunits in prokaryotic proteomes.

### Phylogenetic Tree for Mitochondrial C-I Accessory Subunits Identified in Prokaryotes

To investigate the phylogenetic relationships among the sequences, MSA was conducted using MAFFT with the –localpair option. The resulting alignments were subsequently refined using TrimAl (Capella-Gutiérrez et al. 2009) with the -automated1 parameter to eliminate poorly aligned or ambiguous regions, ensuring the retention of high-confidence sites for phylogenetic inference. A ML phylogenetic tree was constructed using IQ-TREE, employing the LG + F + I + G4 substitution model. Branch support was evaluated through 1,000 ultrafast bootstrap replicates (-bb 1000) and approximate likelihood-ratio tests (-alrt 1000).

## Supplementary Material

evaf154_Supplementary_Data

## Data Availability

The data used in this paper are publicly available. The processed data and scripts used in the manuscript have been uploaded to and are available at the GitHub repository: https://github.com/Anand-Research-Group/Complex-I.
