# Supplementary material for: Hidden Markov Model-Based Prokaryotic Genome Space Mining Reveals the Widespread Pervasiveness of Complex I and Its Potential Evolutionary Scheme
Source: Genome Biol Evol. 2025 Aug 6;17(8):evaf154. doi: 10.1093/gbe/evaf154 (PMC12361115; doi:10.1093/gbe/evaf154)
Supplement: evaf154_Supplementary_Data [file evaf154_supplementary_data.zip › GBE-250350R2-3rd-resubmission-SupplementaryFile.pdf]

Supplementary material for:

Hidden Markov Model-Based Prokaryotic Genome Space Mining Reveals the Widespread Pervasiveness of Complex I and Its Potential Evolutionary Scheme

Akshay Shirsath, Snehal V. Khairnar, Abhirath Anand, Divya M. Prabhakaran, and Amitesh Anand*

Department of Biological Sciences, Tata Institute of Fundamental Research, Mumbai, Maharashtra 400005, India.

*amitesh.anand@tifr.res.in

Content

Figures:

Supp. Figure 1: KDE-based distributions of Nuo-HMMER hits.

Supp. Figure 2: KDE-based distributions of e-values of Nuo subunit search hits.

Supp. Figure 3: Nuo hits distribution by plotting their protein length against the e-value.

Supp. Figure 4: Phylogenetic tree of Nuo subunits, representing one species per genus.

Supp. Figure 5: Phylogenetic tree of Pseudomonadota annotated with accessory subunits of Complex-I.

Supp. Figure 6: The quality of genomes belonging to various Complex I status.

Tables:

Supp. Table 1: Complex I subunits and their homologous proteins.

Supp. Table 2: Custom e-value cutoff for individual Nuo subunits.

Supp. Table 3: Distribution of Complex I variants in archaeal phyla.

Supp. Table 4: Distribution of Complex I variants in Thermodesulfobacteriota.

Supp. Table 5: List of species showing a complete set of Nuo subunits on plasmids.

List of supplementary sheets:

Supp. Sheet 1: List of genomes retrieved from the NCBI genome database

Supp. Sheet 2: List of bacterial species and their genome representation in the analysis

Supp. Sheet 3: List of Nuo subunits hits and their respective ORF

Supp. Sheet 4: List of bacterial species and their associated Complex I variants

Supp. Sheet 5: List of lifestyle information for all species

Supp.Sheet 6: List of bacterial species containing nuo subunit on plasmid

Supp. Sheet 7: List of bacterial species containing mitochondrial Complex I accessory subunit

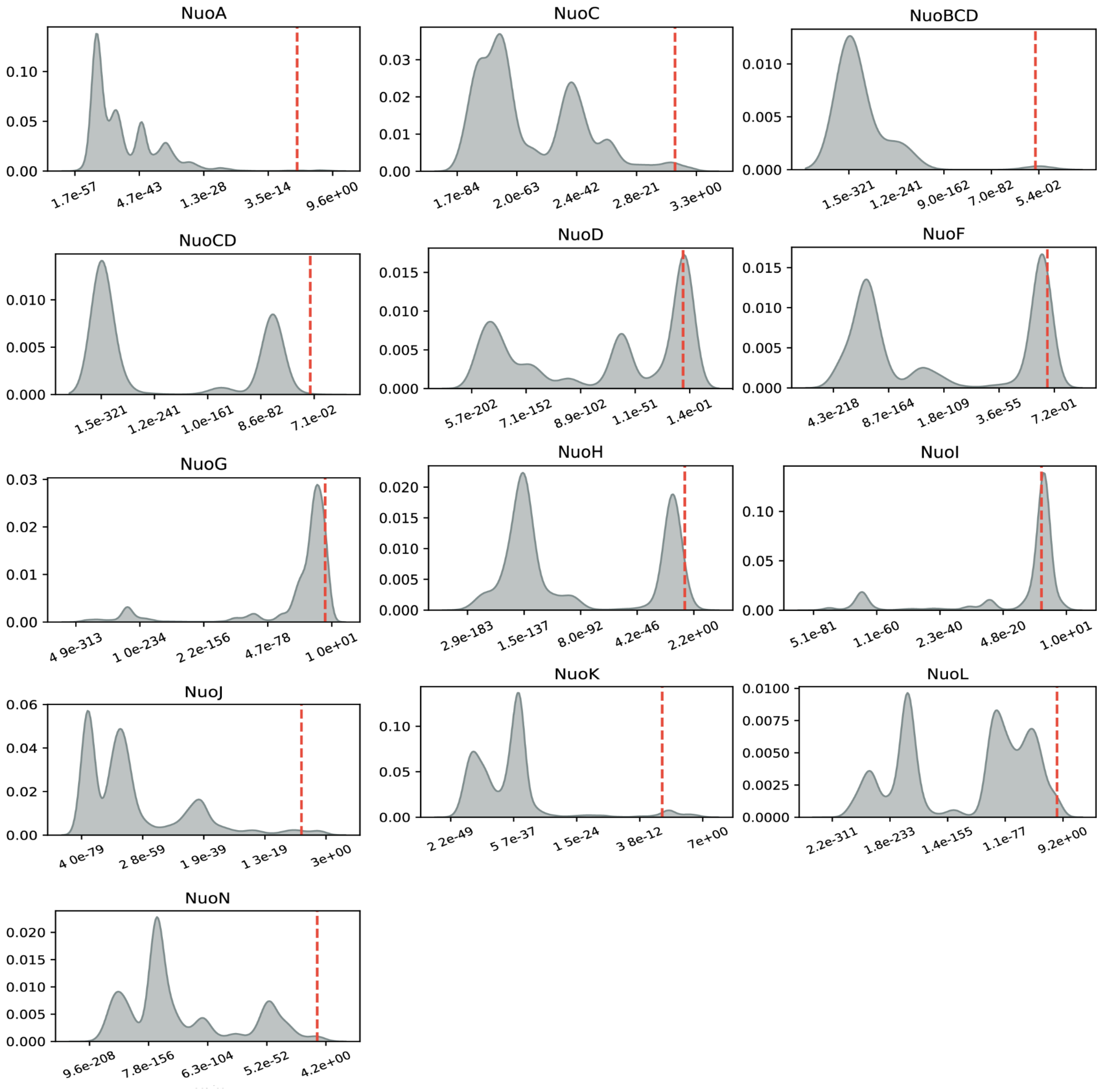

Supp. Figure 1: Kernel Density Estimation (KDE)-based distributions of Nuo-HMMER hits for each Nuo subunit. The x-axis represents e-values, while the y-axis denotes the density of hits. The red dashed line marks the commonly used default threshold of 1e-7 for hit selection in homology-based searches.

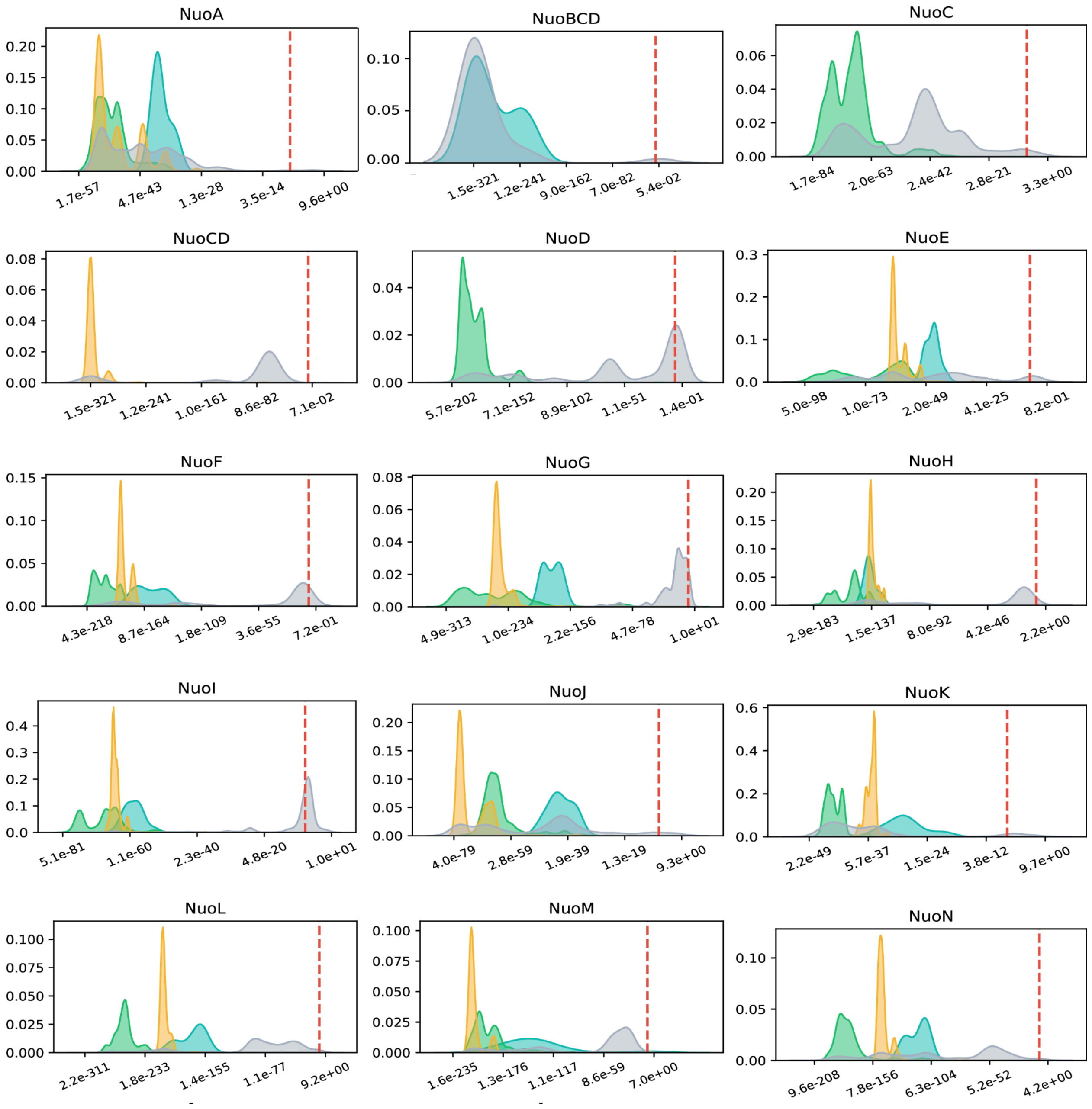

Supp. Figure 2: Kernel Density Estimation (KDE)-based distributions of Nuo subunits search hits' e-values. Hits belonging to the Nuo14 cluster and Nuo13 cluster (with fused CD) are highlighted in green and yellow, respectively. The x-axis represents e-values, while the y-axis denotes the density of hits. The red dashed line marks the commonly used default threshold of 1e-7 for hit selection in homology-based searches.

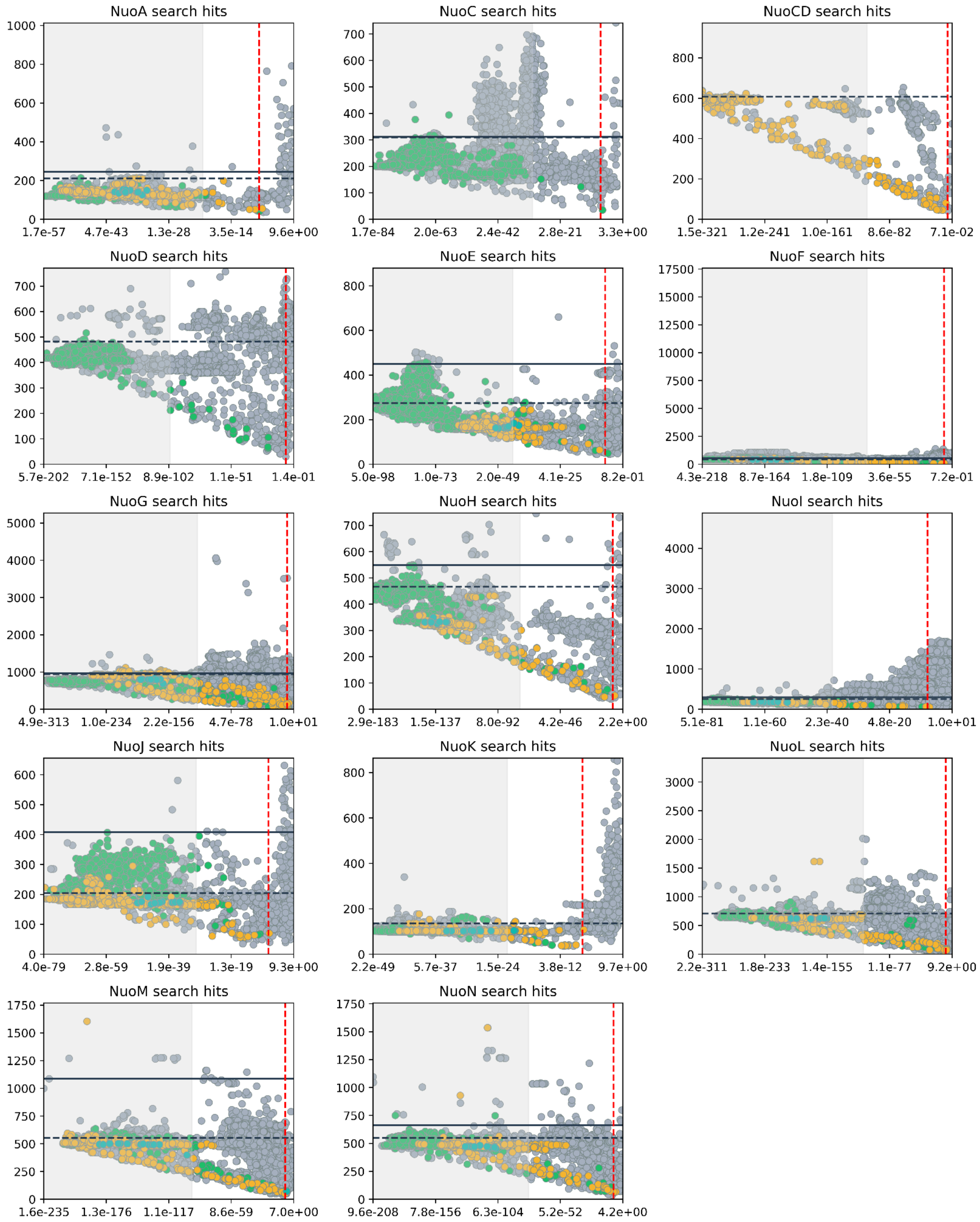

Supp. Figure 3: Nuo hits distribution by plotting their protein length against the e-value.Hits belonging to the Nuo14 cluster and Nuo13 cluster (with fused CD) are highlighted in green and yellow, respectively. The x-axis represents e-values, while the y-axis denotes the protein length of hits. The black dashed line is the Uniprot maximum length for a given subunit. The black solid line is the threshold chosen to remove false hits. The red dashed line marks the commonly used default threshold of 1e-7 for hit selection in homology-based searches. The gray region is a subunit-specific e-value cutoff region.

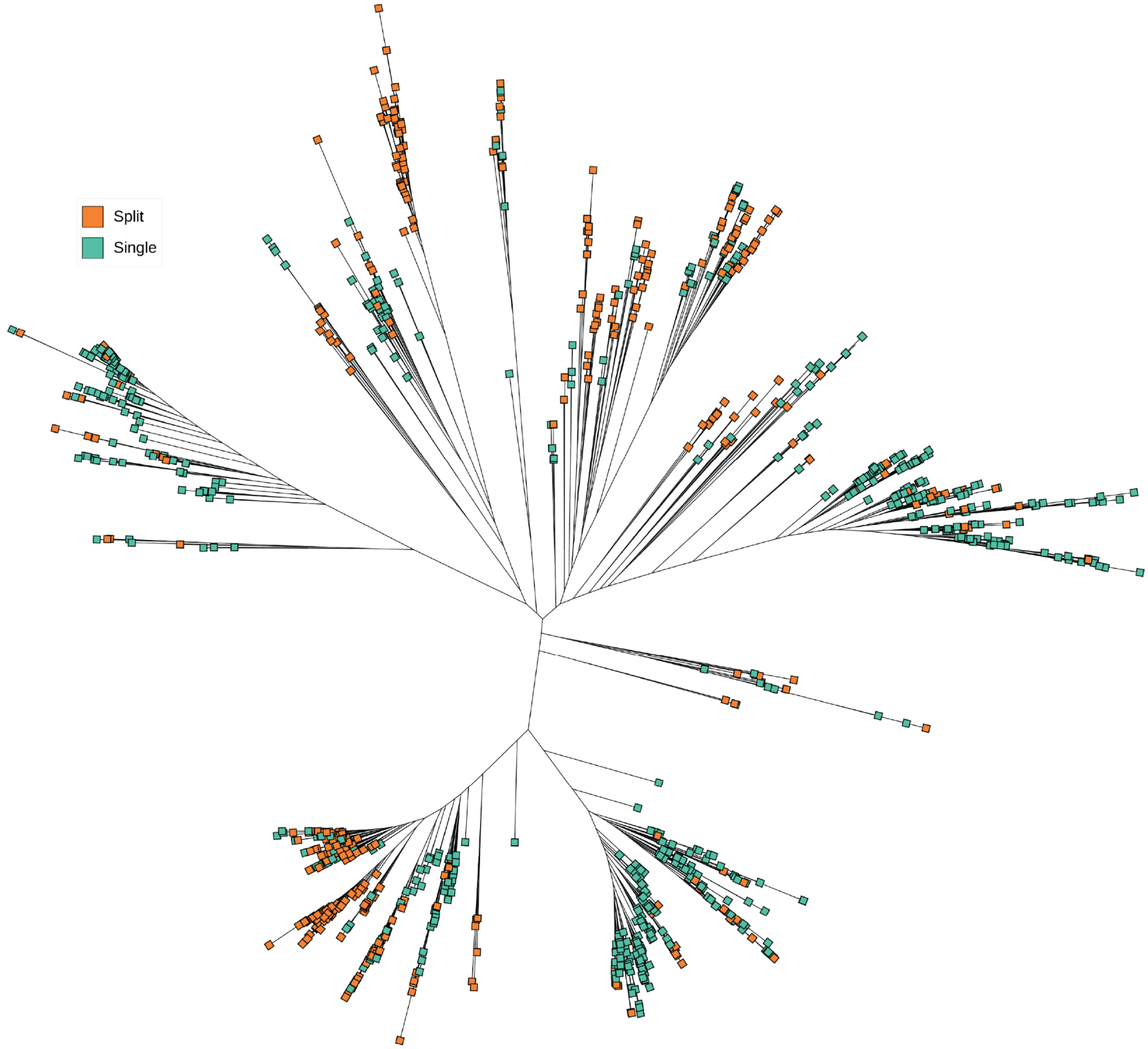

Supplementary Figure 4: Phylogenetic tree constructed from concatenated sequences of Nuo subunits, representing one species per genus. The arrangement of Nuo subunits is indicated by color-filled circles. ‘Single’ denotes all *nuo* genes clustered together within a distance of <250 bases, while ‘Split’ indicates the presence of multiple clusters where *nuo* genes are separated by >250 bases

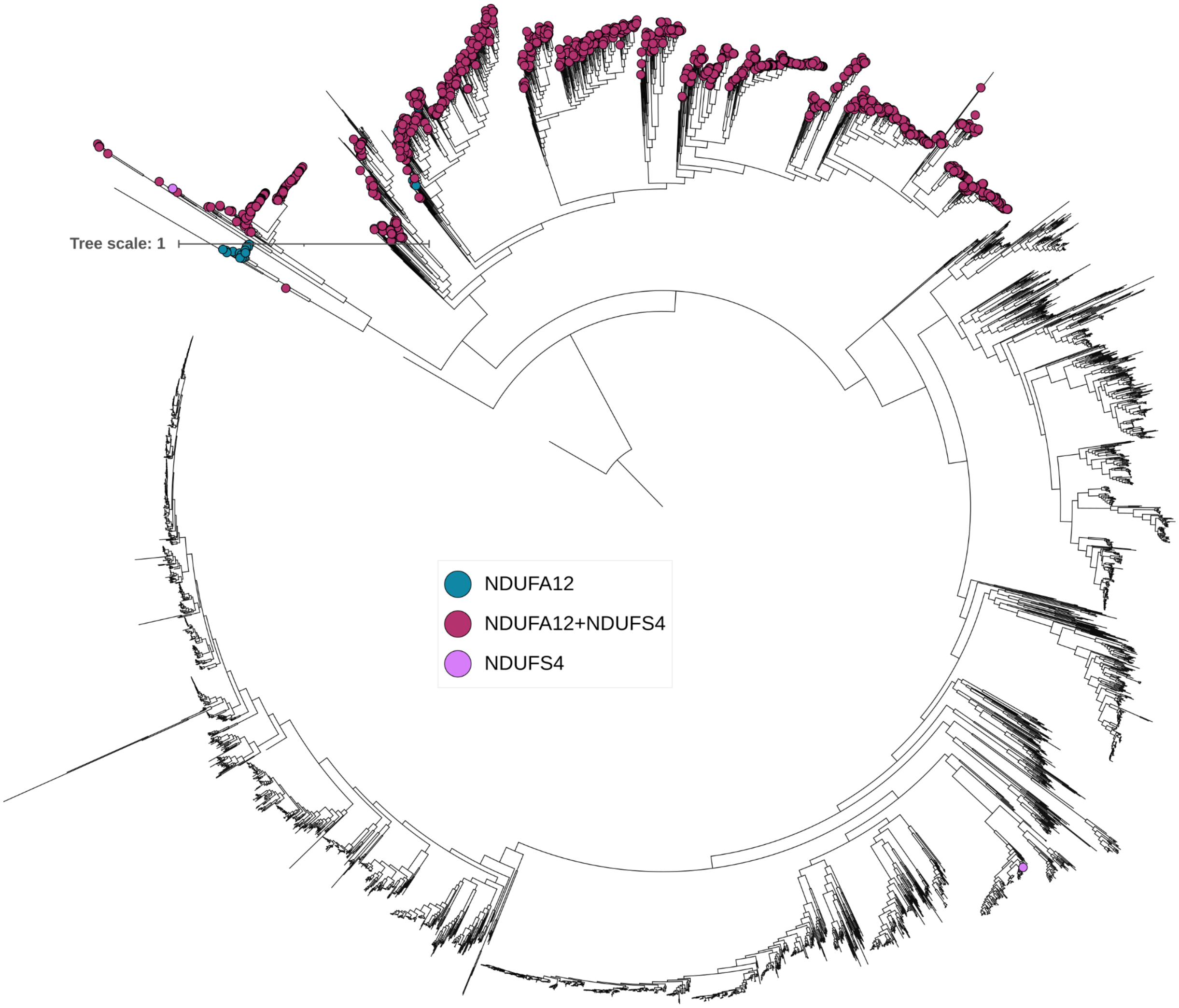

Supplementary Figure 5: Pseudomonadota's (Proteobacteria) circular phylogenetic tree with Complex I accessory subunits annotated. Branch lengths show species separation by corresponding to evolutionary distances. The legend indicates the color-coded presence of accessory subunits. A relative indicator of evolutionary divergence is shown by the scale bar.

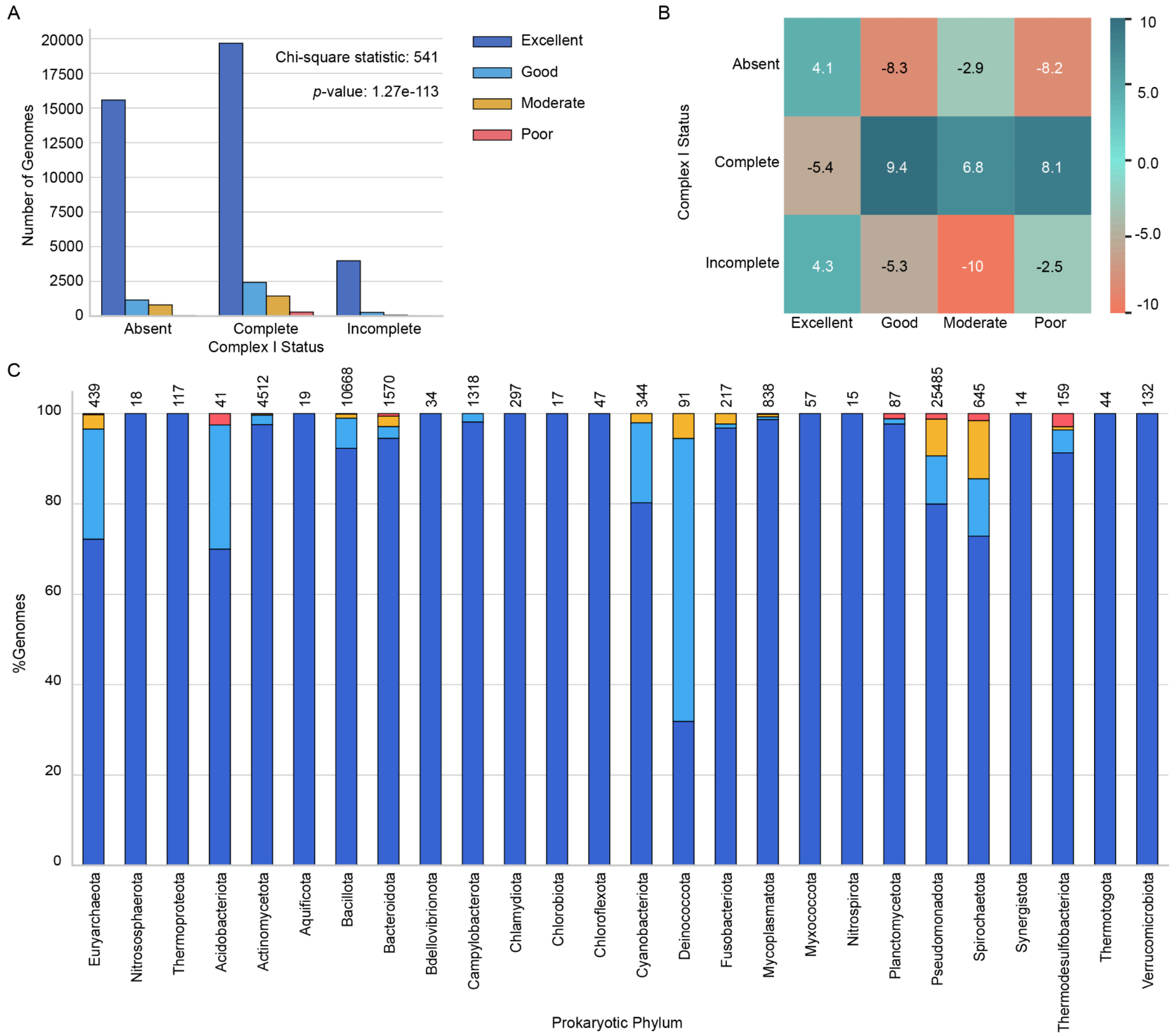

Supp. Figure 6: The quality of genomes belonging to various Complex I status. (A) Bar plot showing the number of genomes classified into each N50 Category (Excellent, Good, Moderate, Poor) for genomes with Complete, Incomplete, or Absent Complex I. The chi-square test statistic and p-value indicate a significant association between Complex I status and assembly quality category. (B) Heatmap of standardized residuals from the chi-square analysis in (A), illustrating the deviation of observed counts from expected counts for each category combination. Positive residuals (teal) indicate over-representation, while negative residuals (coral) indicate under-representation. By cross-tabulating Complex I completeness (Complete, Incomplete, Absent) against assembly N50 Category (Excellent, Good, Moderate, Poor), we observed a highly significant association ($\chi^2 = 540.9$, degrees of freedom = 6, $p \approx 10^{-113}$) (Panel A). Examination of standardized residuals revealed that the genomes lacking one or more Complex I subunits were strongly over-represented in the highest-quality "Excellent" category (Incomplete: +4.3σ; Absent: +4.1σ) and significantly under-represented in all lower-quality categories (Good, Moderate, Poor; residuals ranging from –5.3σ to –10σ) (Panel B). Conversely, genomes with the complete Complex I showed a deficit in Excellent assemblies (–5.4σ) and an enrichment in lower-quality categories (+6.8σ to +9.4σ) (Panel B). (C) Stacked bar plot showing the percentage

distribution of N50 Category within each prokaryotic phylum. Numbers above each bar indicate the total number of genomes assessed per phylum.

Supp. Table 1: Complex I subunits and their homologous proteins

Complex I Subunit (Nuo)	Homologs in Other Complexes	Notes on Homology
NuoA	FpoA[1]	Membrane subunit, conserved in respiratory chains
NuoB	FpoB[1], EchC[2], HycG[3]	Peripheral Fe-S cluster protein
NuoC	FpoC[1], EchD[2], HycE (N-term)[3]	NuoC and NuoD often fused in homologs
NuoD	FpoD[1], EchE[2], HycE (C-term)[3]	Contains NADH-binding domain; fused in some systems
NuoE	FpoE[1], EchA[2]	Part of peripheral arm, involved in NADH oxidation
NuoF	FpoF[1], EchB[2]	Flavoprotein, often conserved across systems
NuoG	FpoG[1], EhaA–E[2], MbhG–M[5]	Contains Fe-S clusters, large and modular
NuoH	FpoH[1], EhaF[2], MbhN[5]	Membrane-bound, central in proton translocation
NuoI	FpoI[1], EhaG[4], MbhO[5]	Peripheral Fe-S subunit, conserved
NuoJ	FpoJ[1], EhaH[4]	Small membrane subunit, variably present
NuoK	FpoK[1], EhaI[4], MbhP[5]	Homologous to smaller membrane subunits
NuoL	FpoL[1], EhaJ–L[4], MbhQ[5], MrpA[6]	Ion-pumping subunit, NuoL-like modules common
NuoM	FpoM[1], EhaM–O[4], MbhR[5], MrpD[6]	Ion-pumping, NuoM homologs widespread
NuoN	FpoN[1], EhaP[4], MbhS[5], MrpD[6]	Ion-pumping, key in proton/ion translocation

References for the table:

[1] Tersteegen, A., Hedderich, R. (1999). F420H2: methanophenazine oxidoreductase (Fpo) complex from *Methanosarcina mazei Gö1*: cloned operon and purification of the active complex. *European Journal of Biochemistry*, 264, 862–871.

[2] Böhm, R., Sauter, M., Böck, A. (1990). Molecular analysis of the Ech hydrogenase operon from *Methanobacterium thermoautotrophicum*. *Molecular Microbiology*, 4, 255–263.

[3]Andrews, S. C., Berks, B. C., McClay, J., Ambler, A., Quail, M. A., Golby, P., & Guest, J. R. (1997). A 12-cistron *Escherichia coli* operon (hyf) encoding a putative proton-translocating formate hydrogenlyase system. *Microbiology*, 143, 3633–3647.

[4] Meuer, J., Kuettner, H. C., Zhang, J. K., Hedderich, R., & Metcalf, W. W. (2002). Genetic analysis of the archaeon *Methanosarcina barkeri* Fusaro reveals a central role for Ech hydrogenase and ferredoxin in methanogenesis and carbon fixation. *Proceedings of the National Academy of Sciences* USA, 99, 5632–5637.
[5] Sapra, R., Verhagen, M. F., & Adams, M. W. (2000). Purification and characterization of a membrane-bound hydrogenase from the hyperthermophilic archaeon *Pyrococcus furiosus*. *Journal of Bacteriology*, 182, 3423–3428.
[6] Ito, M., & Krulwich, T. A. (2017). Evolutionary origins of Na+/H+ antiporter Mrp and its relation to respiratory Complex I. *Frontiers in Microbiology*, 8, 1445. doi:10.3389/fmicb.2017.01445.

Supp. Table 2: Nuo subunit specific e-value Cutoff

Nuo subunit	log10(e-value)	e-value
NuoA	-20	1.00E-20
NuoB	-60	1.00E-60
NuoBCD	-200	1.00E-200
NuoC	-30	1.00E-30
NuoCD	-110	1.00E-110
NuoD	-100	1.00E-100
NuoE	-43	1.00E-43
NuoF	-74	1.00E-74
NuoG	-120	1.00E-120
NuoH	-75	1.00E-75
NuoI	-38	1.00E-38
NuoJ	-30	1.00E-30
NuoK	-22	1.00E-22
NuoL	-110	1.00E-110
NuoM	-95	1.00E-95
NuoN	-78	1.00E-78

Supp. Table 3: Distribution of Complex I variants in Archaea

Phylum	Class	Variant
Euryarchaeota	Methanomicrobia, Halobacteria, Methanobacteria, Thermococci, Archaeoglobi	Incomplete C-I
Nitrososphaerota	Nitrososphaeria	Incomplete C-I
Thermoproteota	Thermoprotei	Incomplete C-I

Supp. Table 4: Class wise distribution of Complex I variants in Thermodesulfobacteriota phylum

Class	Complete C-I (BCD Fused)	Complete C-I (CD Fused)	C-I like (EF/EFG missing)
Desulfarculia	*Desulfoferula mesophila*		
Desulfobaccia			
Desulfobacteria	*Desulfonema limicola*; *Desulfococcus multivorans*; *Desulfosarcina alkanivorans*;		
Desulfobulbia		uncultured *Desulfobulbus* sp.	*Desulfobulbus oralis*
Desulfomonilia			
Desulfovibrionia			*Desulfovibrio desulfuricans*; *Desulfovibrio* sp. G11
Desulfuromonadia	*Syntrophotalea carbinolica* *Desulfuromonas soudanensis* *Syntrophotalea acetylenica* *Syntrophotalea acetylenivorans* *Desulfuromonas versatilis* *Geoalkalibacter halelectricus* *Desulfuromonas sp. AOP6*		*Desulfuromonas acetoxidans*
Syntrophia			
Syntrophobacteria			
Thermodesulfobacteria			*Thermodesulfobacterium commune*; *Thermodesulfobacterium* sp. TA1; *Caldimicrobium thiodismutans*; *Thermosulfuriphilus ammonigenes*; *Thermodesulfatator indicus;* *Thermodesulfobacterium geofontis*; *Thermosulfurimonas marina*

Supp. Table 5: List of species showing a complete set of Nuo subunits on plasmids.

**Species**	**Accession**	**Variation**
Salmonella enterica	NZ_CP148876.1	Complete C-I (CD Fused)
Escherichia coli	NZ_CP141089.1	Complete C-I (CD Fused)
Acinetobacter baumannii	NZ_CP040048.1	Complete C-I (CD Fused)
Acinetobacter baumannii	NZ_CP064203.1	Complete C-I (CD Fused)
Acinetobacter baumannii	NZ_CP104448.1	Complete C-I (CD Fused)
Ralstonia solanacearum	CP088234.1	Complete C-I
Ralstonia solanacearum	NZ_CP115947.1	Complete C-I
Klebsiella pneumoniae	NZ_CP159675.1	Complete C-I (CD Fused)
Klebsiella pneumoniae	NZ_CP129740.1	Complete C-I (CD Fused)
Klebsiella pneumoniae	NZ_CP129873.1	Complete C-I (CD Fused)
Burkholderia vietnamiensis	JAKFAE010000004.1	Complete C-I
Mycobacterium intracellulare	NZ_CP012886.2	Complete C-I
Citrobacter freundii	CP048385.1	Complete C-I (CD Fused)
Klebsiella aerogenes	LR134127.1	Complete C-I (CD Fused)
Komagataeibacter saccharivorans	NZ_CP023037.1	Complete C-I (CD Fused)
Komagataeibacter saccharivorans	NZ_CP036405.1	Complete C-I (CD Fused)
Tsukamurella tyrosinosolvens	LR134465.1	Complete C-I
Legionella adelaidensis	LR134433.1	Complete C-I
Paenibacillus cellulosilyticus	CP054613.1	C-I like (EF/EFG missing)
Salmonella enterica	NZ_CP087508.1	Complete C-I (CD Fused)
Salmonella enterica	NZ_CP087512.1	Complete C-I (CD Fused)
Salmonella enterica	NZ_CP087553.1	Complete C-I (CD Fused)
Sinorhizobium meliloti	CP090106.1	Complete C-I
Sinorhizobium sp. C101	NZ_CP104135.1	Complete C-I
Sinorhizobium sp. M103	NZ_CP104127.1	Complete C-I
Sinorhizobium sp. K101	NZ_CP104131.1	Complete C-I
